# The impact of statins on psychological wellbeing: a systematic review and meta-analysis

**DOI:** 10.1186/1741-7015-10-154

**Published:** 2012-12-03

**Authors:** Adrienne O'Neil, Livia Sanna, Cassie Redlich, Kristy Sanderson, Felice Jacka, Lana J Williams, Julie A Pasco, Michael Berk

**Affiliations:** 1 School of Medicine, Deakin University, Geelong, Victoria, Australia; 2 School of Public Health and Preventive Medicine, Monash University, Melbourne, Victoria, Australia; 3 Unit of Psychiatry, Neuroscience Mental Health and Sensory Organs Department (NeSMOS), Faculty of Medicine and Psychology, Sapienza University of Rome, Sant'Andrea Hospital, Rome, Italy; 4 Masters Programme in Public Health, Department of Clinical Sciences, Social Medicine and Global Health, Lund University, Lund, Sweden; 5 Menzies Research Institute Tasmania, University of Tasmania, Hobart, Tasmania, Australia; 6 Department of Psychiatry, The University of Melbourne, Melbourne, Victoria, Australia; 7 Barwon Epidemiology and Biostatistics Unit, Deakin University, Geelong, Victoria, Australia; 8 ORYGEN Research Centre, Melbourne, Victoria, Australia; 9 Mental Health Research Institute, Parkville, Victoria, Australia

**Keywords:** anti-inflammatory, cytokines, depression, hypercholesterolemia, mood, oxidative, statins

## Abstract

**Background:**

Cholesterol-lowering medications such as statins have anti-inflammatory and antioxidant properties, which may be beneficial for treating depression and improving mood. However, evidence regarding their effects remains inconsistent, with some studies reporting links to mood disturbances. We aimed to conduct a meta-analysis to determine the impact of statins on psychological wellbeing of individuals with or without hypercholesterolemia.

**Methods:**

Articles were identified using medical, health, psychiatric and social science databases, evaluated for quality, and data were synthesized and analyzed in RevMan-5 software using a random effects model.

**Results:**

The 7 randomized controlled trials included in the analysis represented 2,105 participants. A test for overall effect demonstrated no statistically significant differences in psychological wellbeing between participants receiving statins or a placebo (standardized mean difference (SMD) = -0.08, 95% CI -0.29 to 0.12; *P *= 0.42). Sensitivity analyses were conducted to separately analyze depression (n = 5) and mood (n = 2) outcomes; statins were associated with statistically significant improvements in mood scores (SMD = -0.43, 95% CI -0.61 to -0.24).

**Conclusions:**

Our findings refute evidence of negative effects of statins on psychological outcomes, providing some support for mood-related benefits. Future studies could examine the effects of statins in depressed populations.

## Background

Statins (3-hydroxy-3-methylglutaryl coenzyme A reductase inhibitors) are considered first-line agents for treating hypercholesterolemia in the primary and secondary prevention of cardiovascular disease (CVD). Statins lower cholesterol by inhibiting a key enzyme that plays a central role in cholesterol production in the liver, in turn suppressing the inflammatory response to endotoxin and blunting lipopolysaccharide-induced monocyte tissue factor expression [1]. Indeed, the benefits of statin use for individuals with existing coronary disease are well established; a number of past studies have demonstrated their efficacy. The Long-term Intervention with Pravastatin in Ischaemic Disease (LIPID) [2], Scandinavian [3] and Sacks *et al. *[4] studies have all demonstrated positive treatment effects of statins on CVD-related outcomes. However, despite their widespread use, conjecture remains regarding the risk-benefit profile of statins for the primary prevention of CVD, with emerging evidence of adverse effects of rapid lipid lowering on a range of psychological outcomes.

Data from prospective, case-control and longitudinal studies have identified low mood, aggression [5] and suicide [6] as possible side effects of cholesterol-lowering treatment. While the underlying physiology is not fully understood, authors of a review that found an association between low serum cholesterol and suicide suggest that lower serum cholesterol may be associated with reduced serotonin in the brain [7]. Reductions in serotonin have been associated with an inability to suppress aggressive behavior, thus leading to poorer mental health outcomes. Such research has resulted in cholesterol-lowering agents, such as statins, being inextricably linked with adverse psychological outcomes. However, more recent data have challenged this notion.

Statin treatment has now been shown to be associated with improved psychological well-being in individuals with underlying coronary disease, independent of serum cholesterol levels. Young-Xu *et al*. compared 140 patients who had continuous use of statins, with 231 who did not. Statins were associated with a reduced risk of depression (OR 0.63, 95% CI 0.43 to 0.93) and anxiety (OR 0.69, 95% CI 0.47 to 0.99), after controlling for potential confounders [8]. Data from several other studies conducted in hypercholesterolemic [9], cardiac [10,11] and healthy [12] populations have provided further support for this association. A fourfold reduction in risk for depression among individuals taking statins has been observed [10,11]. Using observational data, Pasco *et al*. found that age-adjusted odds ratio for major depressive disorder (MDD) for statin users was 0.13 (95% CI 0.02 to 1.02) [12]. This effect has been demonstrated in both the short term (9-month follow-up) [10] and long term (6-year follow-up) [11].

Using the existing evidence, a review has recently been conducted in an attempt to confirm a directional relationship between statin use and psychological outcomes. While and Keen [13] found conflicting evidence for a relationship between mood and statins, based on the findings of eight studies (prospective, observational, randomized controlled trials (RCTs)). A limitation of this review was that the authors did not meta-analyze the data in order to calculate effect sizes, therefore were unable to define the effects of statins on psychological outcomes. Other recent reviews conducted in the area of statin research that have performed a meta-analysis have failed to shed any light on the effects of statins on psychological outcomes; none have included mental health outcomes [5,14,15]. Thus, the true effect of statins on psychological wellbeing remained largely undetermined.

Using predetermined criteria, our aim was to conduct the first meta-analysis to confirm the impact of statins on psychological wellbeing, in individuals with and without hypercholesterolemia.

## Methods

The literature search identified articles that measured the effects of statins on psychological outcomes (depression, anxiety, mood, psychological distress) in those with and without hypercholesterolemia. The following databases were utilized: Cochrane Central Register of Controlled Trials, PubMed, OVID, Medline, Proquest, CINAHL plus, SCOPUS, Web of Knowledge. Reference lists of relevant reviews and individual studies in this area were manually examined. Search engines (Google Scholar) were used to explore grey literature. No limits on year of publication were set. Studies were limited to those published in English. Bibliographies of extracted references were manually searched for relevant references.

### Selection criteria

Articles included in the review were RCTs meeting the following inclusion criteria: (1) ≥1 placebo/control condition; (2) random assignment to treatment condition of a statin agent (simvastatin, atorvastatin, fluvastatin, lovastatin, mevastatin, pitavastatin, pravastatin, rosuvastatin, cerivastatin) alone/in conjunction with a dietary program; (3) documentation of psychological outcomes (depression, distress, mood) as a primary/secondary endpoint; (4) minimum 2 weeks of treatment (period over which change in mood/depressed state is monitored); and (5) a defined study sample detailing medical status (risk of CVD/hypercholesterolemia, hypercholesterolemia, history of CVD, healthy). Two independent searches that applied this strategy were conducted by the leading authors (AO'N and LS). Results of the searches were then compared and inter-rater reliability assessed. Where consensus for the inclusion of a study was not reached, the third author was consulted.

### Data extraction and validity assessment

To avoid coder bias, data from included articles were extracted by a single investigator and collated into a table for review by the author group. The extraction table included study author, title, journal, drug type and dosage, population, design, assessment points, psychological measure and key findings. Trial quality was assessed according to an inventory guided by Cochrane Collaboration recommendations on evaluating validity. These criteria included: randomization; allocation concealment; blinding; completeness of follow-up; and intention-to-treat analysis. Due to the different measures used to assess depression and mood across studies, estimates were calculated using standardized mean differences (SMD). A random effects model was employed to account for differences in trial designs (for example, crossover, parallel) [16]. Where standard deviations were not reported in the publication (CIs or standard error were instead presented), standard equations for conversion were used to calculate SMD [17]. Where medians and interquartile ranges (IQRs) were reported, medians were substituted for means as recommended by Hozo *et al*., who suggest that when sample size exceeds 25, the median is the best estimator [18]. IQRs were transformed using methods derived from Hozo *et al*. Where a study [19] presented only a between-group change score, normative data for non-psychiatric adult populations were obtained for the depression instrument used in that study (Brief Symptom Inventory - Depression and Anxiety subscale) in the absence of baseline data [20]. In cases where approximations were provided, sensitivity analyses were conducted to determine their influence on overall effect.

Where more than one instrument was used to measure mood state, the instrument most comparable with those used in other included trials was selected. Similarly, where more than one medication arm was included in a trial, the statin agent deemed by the author group to be most comparable with the others included in the review was selected for inclusion in the primary meta-analysis. We re-ran our analysis, imputing data from the second statin arm in each of the multi-arm studies, and it had no influence on results (data not shown). To address heterogeneity among characteristics of study samples (hypercholesterolemia, no hypercholesterolemia), sensitivity analyses were conducted.

### Statistical analysis

The primary meta-analysis measured the effects of statin use on psychological wellbeing (depression and mood) of individuals with or without hypercholesterolemia compared with a placebo, with follow-up periods ranging from 4 weeks to 4 years. Meta-analyses were performed using SMD for continuous outcome measures (based on post-post means), with 95% CIs, using Review-Manager 5 software (RevMan Computer Program. Version 5.1. Copenhagen: The Nordic Cochrane Centre. The Cochrane Collaboration, 2011). We applied Hedges' adjusted *g *to adjust for small sample bias. We explored heterogeneity by calculating the *I*2 statistic and where high *I*2 values were observed, sensitivity analyses was conducted to exclude studies with potential bias or characteristics that were not comparable. *A priori *subgroup analysis estimated the effects of statins on psychological outcomes by length of treatment period, type of statin medication, and psychological outcome (for the purpose of this paper and these analyses, the authors made a distinction between mood and depression in the latter subanalysis for comparability purposes).

## Results

**Figure 1:**
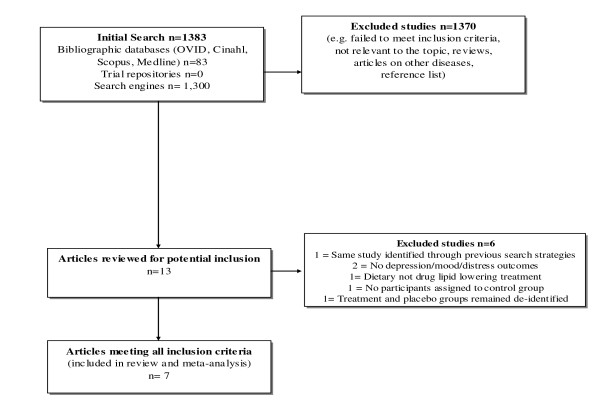
**Study extraction and selection process**.

The literature search was performed from February to March 2012. Using the search terms 'statins' (simvastatin, atorvastatin, fluvastatin, lovastatin, mevastatin, pitavastatin, pravastatin, rosuvastatin, cerivastatin) 'mood' 'distress' 'depress$' 'trial' in bibliographic databases (OVID, Cinahl, Scopus, Medline) and search engines revealed 1383 articles. After reviewing abstracts for relevance, 1370 did not meet inclusion criteria. Reasons for exclusion are displayed in Figure 1, according to Preferred Reporting Items for Systematic Reviews and Meta-Analyses guidelines [21]. Where uncertainty occurred, authors reached consensus by consulting with the third author. Examination of reference lists revealed one potentially relevant article, which was ultimately excluded because treatment and control groups remained deidentified due to the ongoing status of the trial at the time of publication [22]. After completing this process, 13 articles remained. Further examination revealed that two studies did not report depression, anxiety, mood, or distress outcomes; one was the same study identified through previous search strategies; one evaluated a non-pharmaceutical, dietary lipid lowering intervention; one was a study without control condition; and one was the aforementioned paper in which treatment and placebo groups remained deidentified. Thus, these six articles were excluded. After completing this process, seven articles remained for inclusion.

### Study characteristics

Key characteristics are displayed in Table 1. The trials included in the review were published from 1994 to 2006. Studies were conducted in the UK (n = 3) [23-25], Scandinavia (n = 1) [19], Australia/New Zealand (n = 1) [26], and the US (n = 2) [27,28]. All studies defined participants in their samples as those with hypercholesterolemia or elevated serum cholesterol levels with the exception of two, which comprised those with past coronary disease (n = 1) [26], or medically healthy individuals (n = 1) [24].

**Table 1:** Key characteristics of included studies.

Lead author, year, reference	Study design	Agent/dosage	Sample	Measure	Presentation of results	Key finding
Muldoon 2000 [28]	Double-blind, randomized, placebo-controlled trial	Lovastatin (20 mg)/placebo	209 generally healthy adults with LDL cholesterol level of 160 mg/dl or higher were randomized (complete data: drug, n = 98; placebo, n = 96)	Hamilton Depression Rating Scale; NEO-Depression	Means and SDs	24-week treatment of hypercholesterolemia with lovastatin did not cause psychological distress
Wardle 1996 [23]	Randomized, placebo-controlled trial	Simvastatin 20 mg or 40 mg daily/placebo	621 individuals greater higher than average risk of CHD based on medical history (total cholesterol of ≥3.5 mmol/l) were randomized (complete data: drug, n = 334; placebo, n = 157)	Shortened profile of Mood States Questionnaire	Medians and IQRs	152 weeks of cholesterol-lowering treatment did not cause mood disturbance
Hyyppa 2003c	Two separate randomizations, Double-blind, crossover design	Simvastatin 20 mg/day plus dietary intervention/placebo	120 hypercholesterolemic but otherwise healthy middle-aged men (complete data: drug, n = 60; placebo, n = 60)	Brief Symptom Inventory	Mean difference in change scores between groups, SEM	12 weeks of simvastatin resulted in a statistically significant increase in depression
Harrison 1994 [24]	Randomized, crossover design	Sequential placebo, pravastatin 40 mg day/simvastatin 40 mg day, in separate 4-week treatment phases	25 healthy volunteers (17 male, 8 female), average age 23.8 years. Drug, n = 25/n = 25; placebo, n = 25. HADS score for pravastatin was 1.5 (0.6 to 2.4).	HADS, BDI (baseline only)	Means and CIs	No association between simvastatin and depression or anxiety at 4 weeks
Gengo 1995 [25]	Randomized, cross over design	Placebo, lovastatin (40 mg), and pravastatin (40 mg) for 4 weeks	36 patients between the ages of 40 and 60 years with moderate hypercholesterolemia. Drug, n = 24/n = 24; placebo, n = 24. Mood scores for pravastatin 4.8 (6.5).	Profile of Mood States (fatigue/inertia)	Means and SDs	After 4 weeks, no statistically significant differences between lovastatin in changes from baseline were observed on any parameter
Stewart 2000 [26]	Randomized, double-blind, placebo-controlled trial	Pravastatin sodium (40 mg/day) for 4 years	1130 with stable CAD. Drug, n = 559; placebo, n = 571.	General Health Questionnaire (depression and anxiety domains)	Means and SDs	After 4 years no significant increases in self-reported depression, anxiety
Morales 2006 [27]	Randomized, placebo-controlled trial	Simvastatin up to 20 mg/day or placebo for 15 weeks	80 older volunteers, average age 70 years, with high normal/mildly elevated serum cholesterol (placebo, n = 39; drug completers, n = 33)	Center for Epidemiology Studies Depression Scale	Mean and SDs	After 3 months, drug group reported decrease in positive affect

### Psychological wellbeing

Five studies reported depression outcomes at follow-up using Hamilton Depression Rating Scale (clinician administered) [28], Brief Symptom Inventory (self-report) [19], Hospital Anxiety and Depression Scale (HADS) (self-report) [24], General Health Questionnaire - Depression and Anxiety (self-report) [26] and Center for Epidemiology Studies Depression Scale (self-report) [27]. The remaining studies reported mood outcomes using versions of the Profiles of Mood States [23,25]. Four studies measured anxiety using the General Health Questionnaire [26], HADS [24], Brief Symptom Inventory [19], and the Profiles of Mood States [23].

### Intervention

Details of treatment length and dosage are presented in Table 1. Lovastatin (n = 2) [25,28], simvastatin (n = 4) [19,23,24,27] and pravastatin (n = 3) [24-26] were the statin agents evaluated in the seven studies. Two studies included a three-arm design evaluating two statin arms and one placebo arm [24,25]. The remaining five studies comprised a two-arm design (statin versus placebo). Upon consultation with the author group, the type of statin chosen for inclusion in the main effects model for these two studies were those most commonly evaluated in the other studies.

### Trial quality results

We reviewed included studies using a set of quality criteria developed by the Cochrane Centre. Based on this, we concluded that the included studies were of sound methodological quality. Where participant drop out occurred, all studies reported a greater than approximately 80% retention rate. All studies used intention to treat analysis. Details of how investigators and participants were blinded to treatment condition and the process of randomization were unclear in most studies. The key elements of trial quality for each study are presented in Table 2.

**Table 2:** Methodological quality of studies.

Author, year, reference	Agent and dosage	Randomization and concealment	Quality of analysis
Muldoon 2000 [28]	Lovastatin (20 mg)/placebo	Trained data collectors were blinded to subjects' treatment assignment. Method of randomization unclear.	All analyses were conducted on an intention-to-treat basis. 7% attrition rate. Difference in education levels of completers versus drop outs, but no other key characteristics. In several instances, test scores were transformed to ensure normality.
Wardle 1996 [23]	Simvastatin 20 mg or 40 mg daily/placebo	Follow-up data collection was blinded. Method of randomization unclear.	Intention to treat used. Medians and IQRs used. Study had good statistical power to detect a shift in the distribution of the total score of the profile of mood questionnaire.
Hyyppa 2003 [23]	Simvastatin 20 mg/day plus dietary intervention/placebo	All measurements and analyses were performed blinded to the treatment allocation of the participant. Method of randomization unclear.	Intention to treat not mentioned, however attrition rate was 0%. Where necessary, log or square root transformations were applied.
Harrison 1994 [24]	Sequential placebo, pravastatin 40 mg day/simvastatin 40 mg day, in separate 4-week treatment phases	Volunteers were blind to which medication they were receiving. Investigators were blind to drugs administered during the treatment phases but not the placebo washout phases. Method of randomization unclear.	Intention to treat not mentioned, however attrition rate was 0%
Gengo 1995 [25]	Placebo, lovastatin (40 mg), and pravastatin (40 mg) for 4 weeks	Method of randomization or concealment unclear	Intention to treat not mentioned, however attrition rate was 0%
Stewart 2000 [26]	Pravastatin sodium (40 mg/day) for 4 years	Process of randomization or concealment was unclear	Intention to treat used, with the exception of the relation between a change in cholesterol level and psychological outcome. Baseline response rate was 93%. Response rate at follow-up assessments: the response rate was 90% at 6 months, 90% at 1 year, 88% at 2 years, and 77% at 4 years.
Morales 2006 [27]	Simvastatin up to 20 mg/day or placebo for 15 weeks	Blinding: To maintain the double-blind, all medication was prepared in opaque, identical-appearing red-and-blue gelatin capsules, which were sealed in blister cards with each individual dose identified on the packaging by the day and the time it was to be taken. Process of randomization unclear.	Intention to treat analysis used. Mixed effects models used. Where data were not normally distributed, data were transformed/used as binary outcomes. Results were reported in terms of the change in positive affect per day. Given a high degree of colinearity between group assignment and final cholesterol status, these models did not control for treatment group in the analysis of the effect of cholesterol status on outcome. In all, 79% of subjects assigned to placebo and 80% receiving simvastatin completed the study.

### Effect of statins on psychological wellbeing

**Figure 2:**
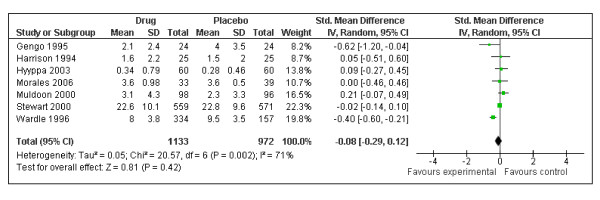
**Overall effect of statin treatment on psychological outcomes of individuals with and without hypercholesterolemia**.

The 7 RCTs included in the analysis represented 2,105 participants (1,133 treatment group versus 972 placebo group). A test for overall effect demonstrated no significant differences in psychological outcomes between participants receiving statins (simvastatin, lovastatin, provastatin) or a placebo (SMD = -0.08, 95% CI -0.29 to 0.12; *P *= 0.42) (Figure 1). Given the high heterogeneity observed across studies (*I*2 = 71%), sensitivity analyses were conducted to separately analyze depression (n = 5) and mood (n = 2) outcomes. This analysis yielded low heterogeneity (*I*2 = 0%) and revealed that statins were associated with significant improvements in mood scores (SMD = -0.43, 95% CI -0.61 to -0.24) (Figure 2), as measured by the Profile of Mood States.

When we compared long-term (3 to 4 years), intermediate (3 to 6 months) and short-term (4 weeks) effects of statin use, no statistically significant differences were observed between groups. When results were explored separately for lovastatin, simvastatin and provastatin, simvastatin was the only agent associated with psychological improvements (SMD = -0.11, 95% CI -0.41 to 0.19), although these effects were not significant.

Further sensitivity analyses were conducted to exclude studies comprising participants with a CVD history [26] or 'healthy' volunteers [24]; this had no impact on results. In a separate analysis, we also excluded studies from which we applied approximations [19,23,24]. The effect of statins on psychological outcomes remained non-significant (SMD = -0.02, 95% CI -0.25 to 0.20).

To explore the effects of statins on anxiety, we included available data from studies that measured anxiety, as distinct from depression (n = 3: one presented data in graphical form [26], therefore were not included in our analysis). This subanalysis compared anxiety data of 419 treatment participants versus 222 control participants, revealing no significant between-group differences (SMD = -0.01, 95% CI -0.17 to 0.16).

## Discussion

The aim of the study was to determine the impact of statins on psychological wellbeing by conducting the first meta-analysis of its kind. Overall, pooled effects of seven RCTs demonstrated no adverse or beneficial effects of statins on psychological outcomes. These findings do not provide support for observational studies that have demonstrated either adverse [5] or protective effects of statins on psychological outcomes in healthy [12] and cardiac populations [10,11]. However, when a sensitivity analysis was conducted to include only those studies evaluating mood, statistically significant effects in favor of treatment were observed. Using Cohen's interpretation of effects, this effect size is considered of medium magnitude [29]. While these findings should be considered with caution due to the limited number of studies included in this subanalysis, they provide some evidence to refute previous suggestions that lipid lowering medications may lead to adverse psychological outcomes. In fact, these data highlight a potential for statins to produce mood-related benefits. This finding is consistent with those RCTs conducted in hypercholesterolemia populations that have observed positive effects on mental health-related quality of life (QoL) [22].

There are several mechanisms by which these effects could be explained. There is now a body of evidence for an etiological role of inflammation and oxidative stress in the pathophysiology of depression [30,31]. As statins have anti-inflammatory and antioxidant properties, it has been suggested that these properties may target the inflammatory and oxidative pathways associated with the pathophysiology of the disorder [32]. Alternatively, because individuals using statins are known to exhibit a decreased risk of cardiovascular events, it is possible that statin-induced mood improvements may be a function of improved QoL or a greater degree of health consciousness for individuals receiving long-term treatment [33].

Large-scale clinical trials with long-term outcomes are required to evaluate the effect of statins on psychological wellbeing of individuals with depression. When conducting our review, we were unable to identify any studies of this type. Because there is some support for the role of statins as a protective mechanism against the development of depression [10], a trial of this nature may offer a novel approach to the treatment and primary prevention of depression through exploring the role of inflammatory processes as a potential therapeutic target. Our findings provide some support for a favorable risk-benefit profile of statins in terms of mood outcomes for those with and without hypercholesterolemia. Statins were not linked to mood-related disturbances, but rather benefits, which may be due to their substantial anti-inflammatory and antioxidative properties. If statin-induced improvements in mood are observed in those with existing depression, the benefits of statins may go beyond that of reducing CVD-related outcomes to depression prevention or therapy. Not only are statins affordable and accessible, but additional benefits exist for those with comorbid conditions that are disproportionally more common in individuals with depression (for example, CVD) [34], because of their impact on shared pathophysiological processes (for example, inflammation) [31].

This meta-analysis has several limitations. Due to the fundamental nature of meta-analyses, where data from independent studies are synthesized, it is acknowledged that unexplained variances may exist, demonstrated by the high degree of heterogeneity observed between studies. For example, it is acknowledged that psychological wellbeing was assessed using different instruments that may measure different constructs. Further, only one study reported using a clinician-administered instrument; the majority of studies used self-report inventories to assess depression and/or anxiety. Therefore, it is recommended that future research pay greater attention to determining the efficacy of statins related to psychological outcomes, employing diagnostic psychiatric interviews to measure these constructs. Although time consuming, this is an accurate method for classifying mood disorders.

Additionally, it is acknowledged that the limited number of studies included in this review may have introduced bias. For example, it is likely that the file drawer effect influenced the findings of this study. This phenomenon sees published studies reporting inflated effect sizes and those yielding negative results remaining unpublished. This is of particular relevance to meta-analyses evaluating the effects of pharmacological interventions. While funnel plots could provide an indication of publication bias, they were not displayed in this paper, as it has been reported that more than ten estimates are required to reliably judge funnel plots [35]. Small sample size further precluded extensive subanalyses of the data, due to limited power (for example, to determine specific effects between those with hypercholesterolemia versus established coronary disease versus healthy participants). Future research comprising a greater number of studies is required to confirm our findings.

## Conclusions

Our findings refute previous evidence of the negative effects of statins on psychological outcomes, and provide some support for mood-related benefits. Further research comprising a greater number of studies is required to confirm the effects of this agent on psychological outcomes. RCTs could further examine the effects of statins in depressed populations.

## Competing interests

FJ has received grant/research support from the Brain and Behaviour Research Institute, the National Health and Medical Research Council, Australian Rotary Health, the Geelong Medical Research Foundation, the Ian Potter Foundation, Eli Lilly and The University of Melbourne and has been a paid speaker for Sanofi-Synthelabo, Janssen Cilag and Eli Lilly. MB has received grant/research support from the NIH, Simons Foundation, CRC for Mental Health, Stanley Medical Research Institute, MBF, NHMRC, Beyond Blue, Geelong Medical Research Foundation, Bristol Myers Squibb, Eli Lilly, Glaxo SmithKline, Organon, Novartis, Mayne Pharma, Servier and Astra Zeneca. He has been a paid consultant for Astra Zeneca, Bristol Myers Squibb, Eli Lilly, Glaxo SmithKline, Janssen Cilag, Lundbeck and Pfizer and a paid speaker for Astra Zeneca, Bristol Myers Squibb, Eli Lilly, Glaxo SmithKline, Janssen Cilag, Lundbeck, Organon, Pfizer, Sanofi Synthelabo, Solvay and Wyeth. LJW has received grant/research support from Eli Lilly, Pfizer, The University of Melbourne, Deakin University and the National Health and Medical Research Council. JAP has received speaker fees from Amgen, Eli Lilly and Sanofi-Aventis and funding from the Geelong Region Medical Research Foundation, Barwon Health, Perpetual Trustees, the Dairy Research and Development Corporation, The University of Melbourne, the Ronald Geoffrey Arnott Foundation, ANZ Charitable Trust, the American Society for Bone and Mineral Research, Amgen (Europe) GmBH and the NHMRC.

## Authors' contributions

AO'N conducted the literature search and quality assessment, performed data extraction and statistical analysis and drafted the original version and subsequent drafts of the manuscript. LS assisted with the inclusion/exclusion criteria, performed literature searching and contributed to the drafting of the manuscript. CR acted as an independent assessor and contributed to the drafting of the manuscript. KS consulted on statistical analysis and critically revised drafts of the manuscript. FJ, LJW and JAP critically revised drafts of the manuscript. MB conceptualized the paper and critically revised drafts of the manuscript. All authors read and approved the final manuscript.

## Pre-publication history

The pre-publication history for this paper can be accessed here:

http://www.biomedcentral.com/1741-7015/10/154/prepub
